# Reduced Absorption Due to Defect-Localized Interlayer Excitons in Transition-Metal Dichalcogenide–Graphene Heterostructures

**DOI:** 10.1021/acs.nanolett.3c01182

**Published:** 2023-06-22

**Authors:** Daniel Hernangómez-Pérez, Amir Kleiner, Sivan Refaely-Abramson

**Affiliations:** Department of Molecular Chemistry and Materials Science, Weizmann Institute of Science, Rehovot 7610001, Israel

**Keywords:** 2D materials, transition-metal dichalcogenides, heterostructures, defects, graphene, excitons

## Abstract

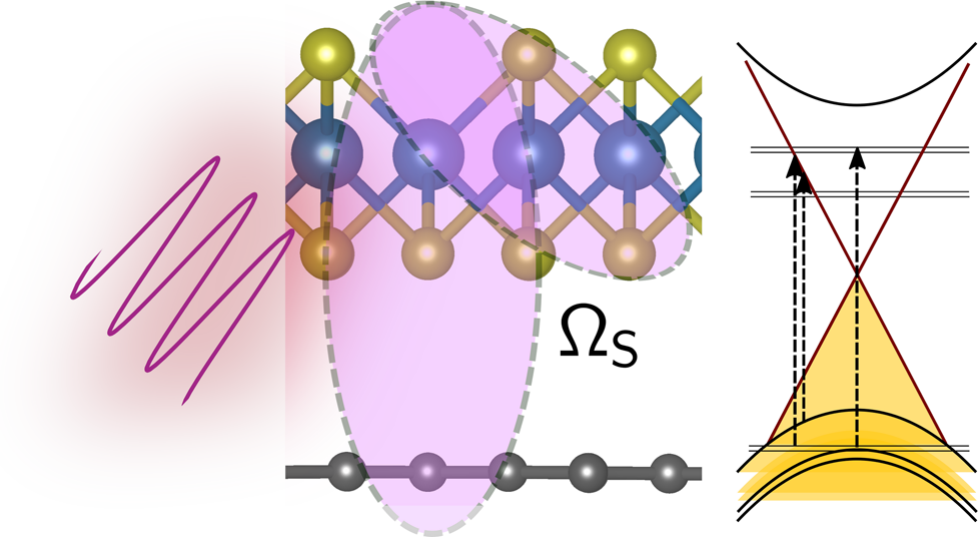

Associating atomic vacancies to excited-state transport phenomena in two-dimensional semiconductors demands a detailed understanding of the exciton transitions involved. We study the effect of such defects on the electronic and optical properties of WS_2_–graphene and MoS_2_–graphene van der Waals heterobilayers, employing many-body perturbation theory. We find that chalcogen defects and the graphene interface radically alter the optical properties of the transition-metal dichalcogenide in the heterobilayer, due to a combination of dielectric screening and the many-body nature of defect-induced intralayer and interlayer optical transitions. By analyzing the intrinsic radiative rates of the subgap excitonic features, we show that while defects introduce low-lying optical transitions, resulting in excitons with non-negligible oscillator strength, they decrease the optical response of the pristine-like transition-metal dichalcogenide intralayer excitons. Our findings relate excitonic features with interface design for defect engineering in photovoltaic and transport applications.

Van der Waals heterostructures,^1−4^ formed by vertically stacking atomically thin two-dimensional layers through weak interlayer interactions, are considered some of the most promising systems for the next generation of ultrathin optoelectronic and photovoltaic high-performance components with tunable properties and tailored functionalities modifiable at the atomic scale.^5−10^ An important example of such heterostructures is the heterobilayer formed by stacking graphene^11,12^ with a monolayer transition-metal dichalcogenide (TMDC) of the type XS_2_, where X is W or Mo.^13−18^ These are type I heterostructures which combine the high carrier mobility,^19^ high thermal conductivity,^20^ and semimetallic character of graphene with pseudospin circular dichroism,^21−23^ large quantum confinement, strong light absorption properties,^24^ and sizable spin–orbit interaction of a direct band gap TMDC.^25,26^

The electronic and optical properties of layered TMDCs and their heterostructures are sensitive to the potential created by defects.^27−31^ In particular, the most abundant and stable point defects in these systems are monatomic chalcogen vacancies.^32−34^ Electron–hole optical transitions between the defect and pristine states are known to produce novel subgap excitonic features^28,35−39^ and form localized excitons that were shown to intrinsically reduce the degree of valley polarization even without additional scattering mechanisms.^28,36,40−42^ Furthermore, changes in the dielectric environment can impact the TMDC intrinsic light emission properties.^43−45^ For instance, interlayer coupling between graphene and TMDC results in a notable quenching of excitonic photoluminescence^46^ or an increase of the exciton line width.^47^ Engineering the exciton spontaneous decay time is also possible by microcavity formation by additional adsorbed layers and the consequent Purcell effect.^48,49^ This effect has been shown to give low-temperature picosecond exciton photoresponses. Therefore, and due to the defect’s ubiquitous nature, a microscopic understanding of the emergent electronic and excitonic properties of TMDC–graphene (Gr) heterobilayers in the presence of vacancies is interesting for dynamic modeling of optoelectronic devices and applications.

In this work, we investigate the electronic and optical properties of WS_2_–Gr and MoS_2_–Gr heterobilayers with monatomic chalcogen vacancies. We employ a GW-BSE approach^50−56^ to compute the many-body electronic properties and optical characteristics and find that due to the combination of screening and strong optical hybridization, absorption resonances of well-known pristine TMDC excitons are strongly quenched in the heterostructure, resulting in substantially altered absorbance properties compared to the pristine TMDC–Gr heterobilayer or defected TMDC without graphene. These pristine-like TMDC “A” and “B” peaks are largely reduced due to the mixing of the optical transitions with both graphene and defect electronic states, and the electron–hole transitions determining their excitonic composition are fundamentally altered. This manifests also in a strong reduction of the excitonic binding energy for the excitons composing those absorption peaks. In addition, Fermi-level alignment of the defect transition levels and the magnitude of the spin–orbit interaction, determined by the choice of TMDC, lead to substantial changes in the heterostructure optical properties. We obtain the intrinsic radiative rates for excitons with the largest binding energy, which create strongly mixed subgap resonances and show that the associated inverse rates are comparable to those calculated for pristine TMDC monolayers.

We employ a commensurate supercell composed of 4 × 4 WS_2_ (respectively MoS_2_) and 5 × 5 graphene elementary cells; see Figure 1a and the Supporting Information (SI). We consider a vacancy concentration of ∼3%, corresponding to an isolated monoatomic sulfur vacancy per supercell, located at the opposite side of the graphene layer with sufficiently small interactions between defect sites at neighboring periodic replicas of the supercell.^17^ We first perform a geometry optimization of the supercell atomic positions, keeping the supercell volume constant (see ref (17) and the SI). This optimization reduces the nearest-neighbor bonds close to the vacancy (which shrinks and strains the TMDC lattice) as well as the interlayer distance between the TMDC and the graphene layer. Using DFT as a starting point (with the PBE functional^57^), we perform a one-shot GW (G_0_W_0_) calculation for each TMDC–Gr heterobilayer (see computational details in the SI). Figure 1b shows the DFT and GW energies at the point K̅ of the supercell Brillouin zone in an energy level diagram. As expected based on previous studies,^28,58,59^ GW quasi-particle corrections increase the gap in both systems, qualitatively, conserving the DFT picture for these heterobilayers.^17^ There are four spin–orbit split defect states shifted upon the GW calculation to higher energy with respect to the Fermi level. Far from the Fermi level, we find the valence band splitting due to spin–orbit interaction to be ∼460 meV for WS_2_–Gr and 150 meV for MoS_2_–Gr, which is consistent with 425 ± 18 and 170 ± 2 meV obtained from high-resolution ARPES measurements.^26^ The dielectric screening also shifts the pair of occupied defect states to lower energies (by ∼400–450 meV for WS_2_–Gr and ∼400 meV for MoS_2_–Gr). Simultaneously, the pristine-like band gap of WS_2_ increases from 1.69 eV at the DFT level to 2.20 eV at the GW level. Similarly, the pristine-like band gap of MoS_2_–Gr changes from 1.73 to 2.19 eV. These values reflect a significant renormalization of the TMDC band gaps compared to the monolayer case, as expected due to the quasi-metallic character of graphene with GW corrections including image charge effects.^59^ Our GW results are in agreement with previous calculations with a reported band gap reduction of ∼300–350 meV^59,60^ compared to each pristine counterpart.^58^ They are also consistent with the experimental values of the quasi-particle band gap found in MoS_2_–Gr heterostructures, reported to be ∼2.0–2.2 eV,^61,62^ and in WS_2_–Gr, which is in the range ∼2.0–2.3 eV.^14,63,64^

**Figure 1:**
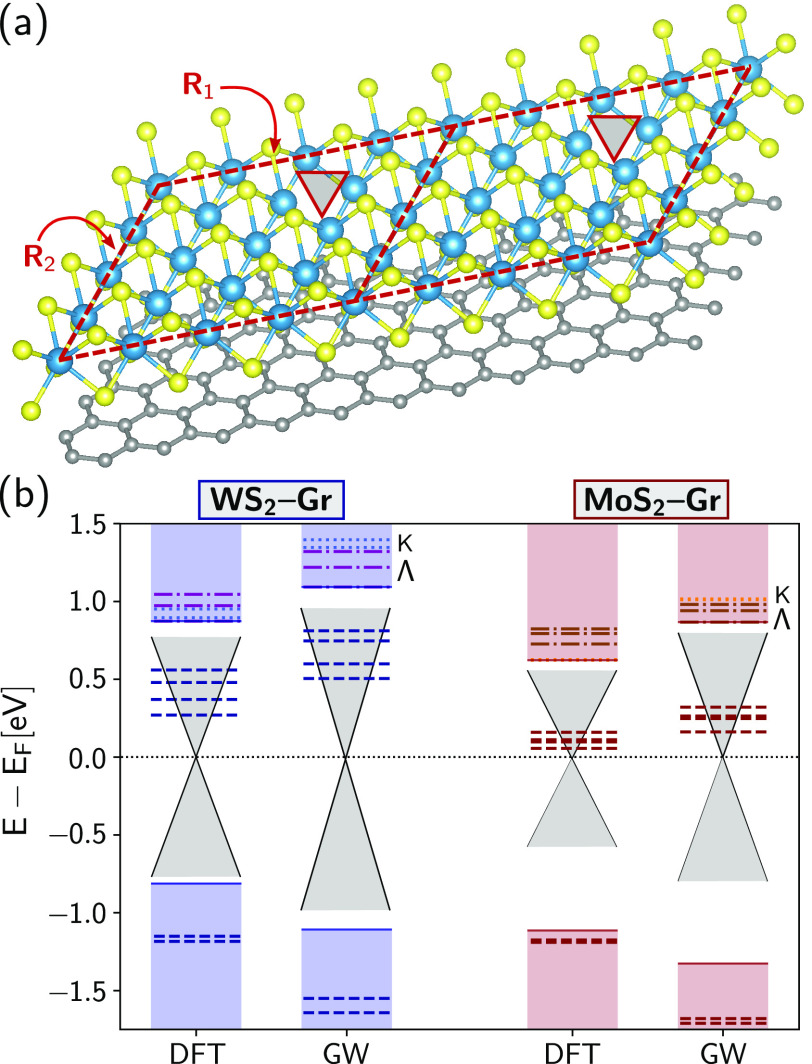
(a) Schematics of the defected WS_2_–Gr heterobilayer. Each supercell (two are shown here) forms a parallelepiped with lateral boundaries marked by the straight red lines (in-plane supercell lattice vectors are denoted by **R**_1_ and **R**_2_). The monoatomic chalcogen vacancy position, located opposite to the graphene layer, is indicated by a red triangle. (b) DFT and GW calculated valence and conduction band energies at the K̅ point. The blue bars on the left-hand side correspond to WS_2_–Gr and the red bars on the right-hand side to MoS_2_–Gr. The dashed lines denote the defect energy levels, and the solid lines mark the top of the pristine TMDC valence and conduction bands. Energies are related to the Dirac point of graphene, *E*_F_, which sets the Fermi energy of the system. The gray sketch of the Dirac cone represents the region of the TMDC pristine band gap occupied by graphene. Conduction levels with predominantly Λ nature are represented by the dashed-dotted lines, with those with predominantly K nature represented by dotted lines.

Next, we examine the excitonic properties of defected WS_2_–Gr and MoS_2_–Gr heterobilayers using the Bethe–Salpeter equation^54,55^ within the Tamm–Dancoff approximation (see the SI). We show in Figure 2, top panel, the absorbance of WS_2_–Gr as well as its decomposition into intralayer graphene, intralayer TMDC, and interlayer contributions (see the SI for the case of MoS_2_–Gr). At low optical energies, intralayer graphene electron–hole excitonic features are found to be the most prominent. Graphene electronic intraband transitions (not considered in our calculations, as well as temperature effects) are known to dominate this regime,^65^ which is marked by a dashed gray rectangle. The resonant peaks are a consequence of the finite **k**-grid sampling of the graphene Dirac cone, and therefore, the absorption in this region is actually continuous in the dense grid limit. In the high infrared spectral range, excitonic peaks corresponding to interlayer graphene–defect and graphene–pristine optical transitions appear at higher energies while graphene intralayer contributions become less relevant. At optical energies ≳2.0 eV, intralayer TMDC contributions (in the form of defect–defect, defect–pristine, and pristine–pristine band transitions) become the dominant features of the spectrum over the interlayer contributions. Out of all optical transitions sampled for the examined **k**-grid and energy range, 0.4% belong to intralayer graphene, while 62% correspond to intralayer TMDC transitions, with the remaining 37% representing a large degree of interlayer mixing.

**Figure 2:**
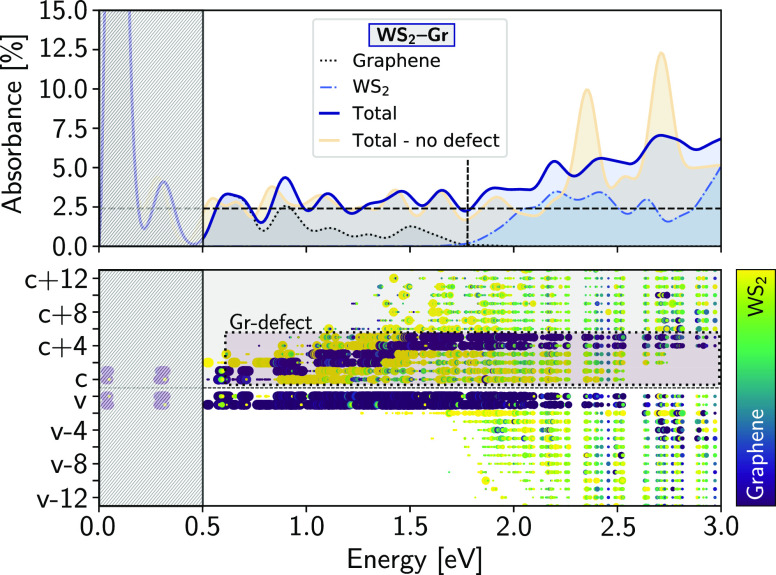
Absorbance and exciton contributions for the defected WS_2_–Gr heterobilayer. (top) Absorbance calculated along one of the main in-plane polarization directions as well as its decomposition into intralayer graphene and WS_2_ contributions (interlayer contributions are read from the difference between the three traces). For comparison, we also show the absorbance of the pristine WS_2_–Gr heterobilayer. The dashed horizontal black line marks the 2.4% universal limit of graphene absorbance at the infrared energies. The shaded box represents the estimated range for which we expect a smooth and monotonic spectrum dominated by graphene (instead of resonances resulting from finite **k**-grid sampling). The vertical dotted line marks the optical ranges below which excitons are dominated by defect-graphene subgap transitions to a range where excitons present larger intralayer TMDC composition. (bottom) For each exciton composing the absorbance resonances, we represent the contribution of each electron and hole band. Each dot corresponds to the band contribution to a given exciton summed over all **k** points (only bright contributions whose oscillator strength are >5 a.u. are shown). For clarity, all dots with values ≥10^3^ a.u. have the same area. The color code corresponds to the layer composition of each contribution, and the dotted box marks the graphene-defect empty bands.

The spectral absorbance of graphene for infrared light is almost constant at 2.4%.^65−71^ This limit is represented by a dashed horizontal line in Figure 2. We find that absorbance resonances in the optical range between ∼1.0 and 1.6 eV oscillate around values larger than the graphene infrared constant limit. This is associated with excitons that involve defects and should persist in the dense grid limit. The computed absorbance values in this range are also consistent with those calculated for defected MoSe_2_ in the absence of graphene.^28^ To further validate our findings, we compare the absorbance spectra of WS_2_–Gr with and without vacancies. We observe that, unlike the defected heterostructure, the absorbance of the pristine heterobilayer oscillates around the graphene limit in that energy range, supporting our previous conclusion. In the visible range (i.e., ∼1.6–3.2 eV), experiments on WS_2_ have reported a red shift and a significant reduction of the exciton peak area upon graphene stacking.^47,72^ These effects are typically attributed to both changes in the dielectric environment upon the heterostructure formation and possible interfacial charge transfer.^73^ Our results demonstrate that within the exciton basis set, optical transitions at both layers mix already upon light absorption. In defected WS_2_–Gr heterobilayers, we observe a reduction of the strength of the pristine-like TMDC resonances in addition to the substantial interlayer mixing in the subgap optical region dominated by transitions to the defect states. This effect is attributed to the strong optical mixing of TMDC and graphene, which results in additional interlayer optical transitions. These occur within the pristine gap at many different **k**-points due to the dispersive nature of the Dirac cone and yield a redistribution of the oscillator strength from the original pristine transitions to the defects. These combined effects of the graphene and vacancies quench the pristine-like “A” and “B” resonances^74,75^ and also broaden them.^41^ As a consequence, they are no longer as dominant in the spectrum. Moreover, the composition of the absorption peaks, clearly defined at ∼2.2, ∼2.4, and ∼2.7 eV, also changes drastically compared to the expectation for the pristine or defected TMDC monolayers (see the SI).

To further understand this effect, we show in Figure 2, bottom panel, the contribution of each band to the exciton (similarly to our previous analysis of exciton state mixing in defected systems^28,36,76^). Each excitonic state, |Ψ^*S*^⟩, defined by its wave function amplitude *A*^*S*^_*vc***k**_ and energy Ω_*S*_, is represented by a column of dots whose area is proportional to ∑_*v***k**_ |*A*_*vc***k**_^*S*^|^2^, for each electron band (*c*), and ∑_*c***k**_|*A*_*vc***k**_^*S*^|^2^, for each hole band (*v*). The color of the dot represents the layer from which *c* or from which *v* the transitions occur. We observe intralayer graphene optical transitions in the low energy region (≲0.5 eV), while excitonic resonances with interlayer character appear only above 0.5 eV. The dispersive nature of graphene can be seen from the increase in the conduction band number of graphene with energy. As expected, the quenched high-energy resonances show significant contribution of mixed TMDC and graphene optical transitions; thus, although they appear in similar positions, they no longer possess true “A” and “B” characters (see the SI). We note that while the increase of the dielectric screening by the surrounding media and the reduction of the absorbance is expected,^64^ here the effect is further pronounced due to the electron–hole defect and nondefect mixing in the subgap region.

The binding energy of the exciton quantifies how strongly bound the electrons and holes participating in the excitation are. Intralayer graphene excitonic features have vanishing small binding energies (∼0–1 meV, see also refs (65 and 77)) despite their strong oscillator strength. This differs significantly from pristine or encapsulated TMDC excitons, which have a large oscillator strength and binding energies. In particular, experimental estimations of the binding energies are 0.3–0.4 eV for MoS_2_ and 0.3–0.7 eV for WS_2_ pristine “A” excitons.^78^ Theoretical predictions give 0.6 eV for MoS_2_ and 0.2 eV for WS_2_ pristine “B” excitons.^75^ In Figure 3a, we present the exciton energy as a function of the binding energy for WS_2_–Gr (see the SI for MoS_2_–Gr). The binding energy is computed from the energy difference between the free and bound electron-hole pairs composing each exciton (see SI). For excitons in the optical region where the pristine “A” and “B” resonances would be expected, we observe that excitons have substantially lower binding energies compared to the pristine or encapsulated counterparts, as well as substantially smaller oscillator strengths. We attribute this to a redistribution of the oscillator strength due to the combined effect of substantial hybridization of the defect and nondefect electron–hole transitions with graphene, as well as the small binding properties of excitons in graphene resulting from its quasi-metallic nature at low energies. Importantly, we also find excitons (with oscillator strength in the range 10^–2^–1.0 a.u.) in the optical region 1.5–2.0 eV with a binding energy comparable to that of pristine excitons in the absence of a graphene layer. These excitons result from intralayer optical transitions to defect states and interlayer graphene–defect transitions. To gain insight into the nature of these excitons, we show in Figure 3b the **k**-space distribution for a representative case marked by a circle in Figure 3a. It is worth noting that their degree of localization cannot be used to infer the strength of the binding, as excitons with similar binding may exhibit optical transitions in very different regions of the Brillouin zone due to the dispersive nature of the graphene bands and the delocalization of defect states in **k**-space. The right-hand side of Figure 3b displays the optical transitions at selected **k**-points, supporting our analysis that this exciton is formed by a combination of defect–defect (notably at K̅), graphene–valence, and graphene–defect transitions (for the **k**-point noted as k*).

**Figure 3:**
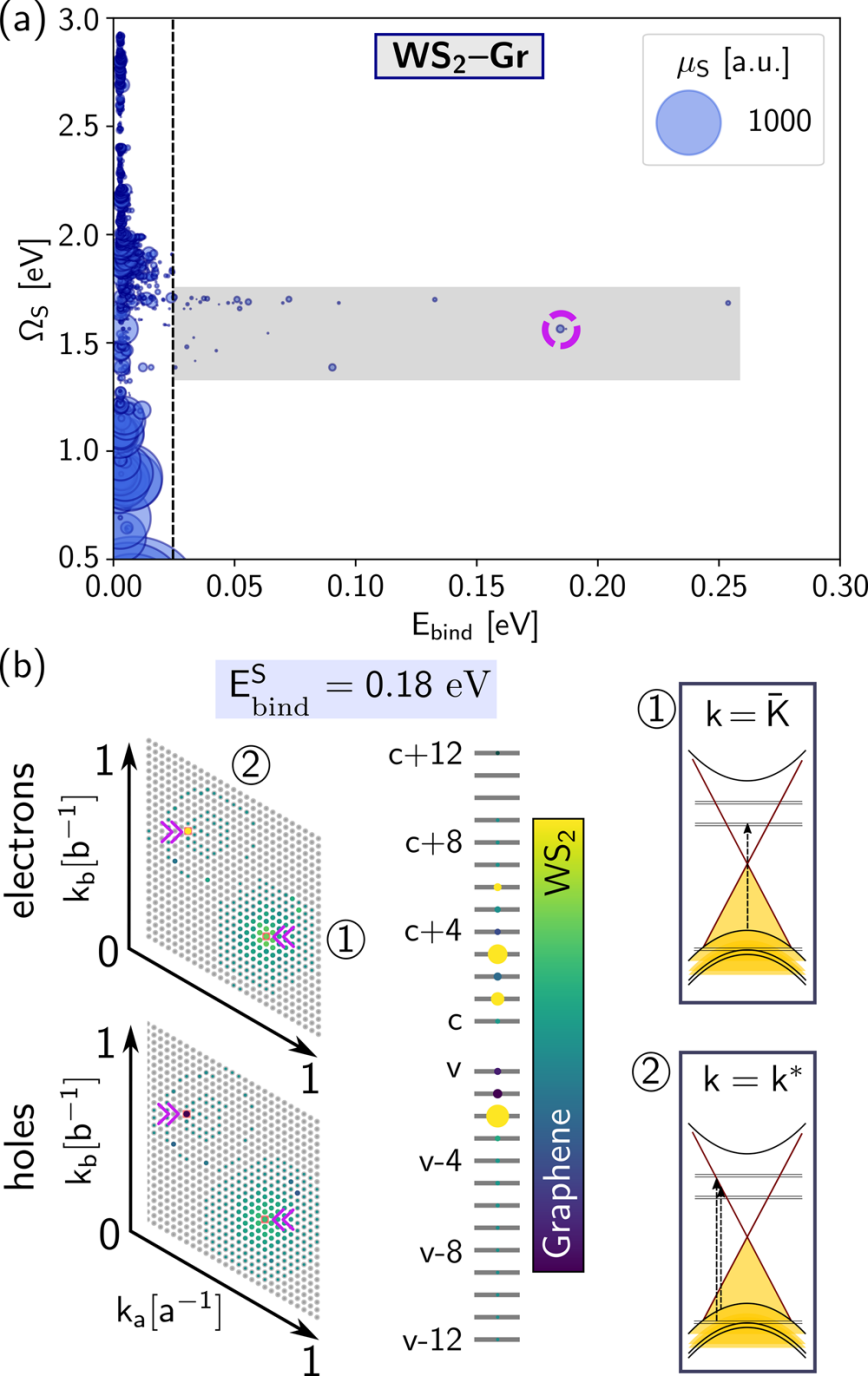
(a) Exciton energies for WS_2_–Gr, Ω_*S*_, as a function of their binding energy, *E*_bind_. Only excitons for which *E*_bind_ > 2.5 meV are shown (∼8000 out of 142884 excitons for our **k**-grid sampling and bands). The size of each dot is proportional to the oscillator strength, μ_*S*_. Bright excitons, in particular those dominated by intralayer graphene transitions, have very small binding energies (smaller than the thermal energy at room temperature, marked by a dashed black vertical line). Excitons within the energy range where the pristine “A” and “B” features would be expected are dark and have a very small binding energy. The gray rectangle corresponds to excitons with binding energies larger than 25 meV. (b) Brillouin zone exciton distribution and transition band diagram for the exciton marked with a purple circle in (a). The top Brillouin zone corresponds to transitions to the conduction bands, and the bottom one corresponds to transitions from the valence bands. On the right-hand side schematics, we show selected transitions marked by ① and ② at the **k**-points indicated by purple arrows.

Finally, we relate our findings to the intrinsic radiative decay rates of zero-momentum excitons, which can be computed from the GW-BSE oscillator strength and excitation energy.^79−81^ We consider the inverse rate, which scales as γ_*S*_^–1^ ≔ τ_*S*_ ≈ Ω_*S*_/μ_*S*_. This rate accounts only for part of the radiative line width, as other contributions, e.g. electron–phonon and exciton–phonon terms, are not included, and is useful to evaluate the significance of the oscillator strength. Our analysis reveals that the inverse rates for the excitons with binding >50 meV can be as large as τ_*S*_ ≈ 0.1 ps for both heterobilayers. However, depending on the oscillator strength, they can be shorter, even as small as τ_*S*_ ≈ 0.1 fs for MoS_2_–Gr (see the SI). Intralayer graphene excitonic features, which have significantly small binding, have substantially larger intrinsic rates due to their large optical transition dipole. Dark interlayer excitons with large binding have larger inverse rates, as large as ∼100 ps for WS_2_–Gr, due to the smaller oscillator strength. For pristine WS_2_–Gr bright “A” and “B” excitons in the TMDC layer, τ_*S*_ can be even shorter, essentially due to the increased oscillator strength (between 2 and 3 orders of magnitude compared to the defect-related excitons, see the SI) which yields τ_*S*_ ≈ 10^–4^–10^–5^ fs. We note that compared to pristine TMDCs,^80^ graphene adsorption has a strong impact on τ_*S*_, which only become comparable again to the pristine ones in the presence of defects due to the strong exciton hybridization of the graphene and the subgap vacancy-related features. Furthermore, charge transfer times of photocarriers at TMDC–graphene interfaces,^13−15,82,83^ where single-particle defect tunneling is understood to be the dominating coherent transport channel,^14,17^ can be of a similar order of magnitude. In this scenario, defects slow down coherent charge transfer due to relatively small interlayer tunneling. Interestingly, in the presence of graphene, defects optically enhance transitions associated with them, resulting in excitons with significantly higher oscillator strength, compared to the reduced oscillator strength of the original pristine-like “A” and “B” TMDC excitons.

In conclusion, we have studied the electronic and optical properties of WS_2_–Gr and MoS_2_–Gr heterobilayers with chalcogen vacancies by employing first-principles many-body perturbation theory. We find that strong hybridization of the defect states with graphene gives rise to subgap features, which manifest as strong resonances in the optical absorbance spectrum, while quenching the “A” and “B” pristine exciton peaks originally coming from intralayer TMDC transitions. These altered absorption features may be used to extend the functionality in the infrared of solar cells. We have analyzed the stability of the excitons and found a strong reduction of the binding energy for those TMDC excitons, while strongly hybridized interlayer and defect-dominated excitons have binding energies up to ∼250 meV. We computed the intrinsic radiative decay rate of these excitons and found inverse rates of up to 0.1 ps. Overall, our results demonstrate how point-like defects can be used to design optical features in graphene-based van der Waals heterostructures, where excitons inherit properties from two well-distinct layers in a nontrivial way, pointing to the relevance of a first-principles understanding of many-body effects in the description of these systems for transport and potential optoelectronic applications.

## Supporting Information Available

Computational details and methods, additional results, convergence checks, absorbance, and additional results for MoS_2_–Gr, wave function density of relevant conduction states, exciton reciprocal space composition for relevant energies in the WS_2_–Gr spectra, and additional figures (PDF)
