# Supplementary material for: Reduced Absorption Due to Defect-Localized Interlayer Excitons in Transition-Metal Dichalcogenide–Graphene Heterostructures
Source: Nano Lett. 2023 Jun 22;23(13):5995–6001. doi: 10.1021/acs.nanolett.3c01182 (PMC10347701; doi:10.1021/acs.nanolett.3c01182)
Supplement: Supplementary file 1 — nl3c01182_si_001.pdf [file nl3c01182_si_001.pdf]

Supporting Information

Reduced absorption due to defect-localized interlayer excitons in transition metal dichalcogenide–graphene heterostructures

Daniel Hernangómez-Pérez,[1] Amir Kleiner,[1] and Sivan Refaely-Abramson[1]

[1]*Department of Molecular Chemistry and Materials Science, Weizmann Institute of Science, Rehovot 7610001, Israel*

Keywords: 2D materials, transition-metal dichalcogenides, heterostructures, defects, graphene, excitons

Email: daniel.hernangomez@weizmann.ac.il
sivan.refaely-abramson@weizmann.ac.il

CONTENTS

I. Computational details and methods 2

II. Additional results 7

III. GW and absorption convergence checks 9

IV. Absorbance spectra and defect resonances in MoS_2-graphene 12

V. Wavefunction densities of relevant conduction bands 13

VI. Defect-induced exciton hybridization 15

VII. Additional figures 17

References 20

I. COMPUTATIONAL DETAILS AND METHODS

Geometry. As a starting point for the supercell optimization in the presence of the vacancy (see details below), we consider the geometry of a pristine heterobilayer with a lattice parameter whose length corresponds to an average nearest-neighbor metal-metal distance (equal to the in-plane monolayer TMDC lattice constant) of $\bar{d}_{\mathrm{X-X}} = 3.15\,\text{Å}$ both for MoS_2 and WS_2. This value is almost equal to the experimental monolayer lattice parameter of both TMDC (MoS_2; 3.15 Å [1] and WS_2; 3.153 Å [2]). As stated in the main text, the supercell is composed of 4×4 TMDC unit cells and 5×5 graphene unit cells. Therefore, it is made of 97 atoms and possesses a rhomboedral shape with in-plane lattice vectors of length $|\mathbf{R}_{1,2}| \simeq 12.6$ Å (see Fig. S1). This supercell size allows us to describe isolated vacancies, within a defect density of $\sim 3\%$, at least one order of magnitude larger than the typical intrinsic vacancy concentration[3]). The vacuum distance between the periodic replicas in the out-of-plane direction was taken to be ~ 10 Å and the average interlayer distance within the supercell was $\bar{d}_{\mathrm{inter}} = 3.43$ Å both for Mo and W.

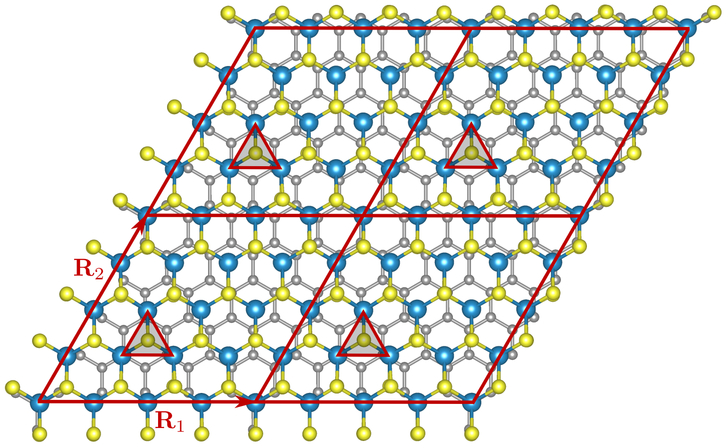

Fig. S1. Top view of the WS_2–Gr supercell. Four supercells are shown, each supercell forms a parallelepiped whose lateral boundaries are given by the straight red lines (the in-plane supercell lattice vectors are labeled as $\mathbf{R}_1$, $\mathbf{R}_2$). The chalcogen vacancy, located in the TMDC layer on the opposite side of the graphene layer, is indicated by a red triangle.

Density functional theory. The DFT calculations were performed employing the implementation of DFT of Quantum Espresso.[4,5] We used the non-empirical PBE generalized gradient approximation for the exchange–correlation functional[6]. We employed a

plane-wave basis set and included spin-orbit interaction by means of full relativistic norm-conserving pseudopotentials[7]. We considered a basis cut-off of 50 Ry for both TMDC–Gr interfaces. The self-consistent charge density was converged within a $6 \times 6 \times 1$ $\mathbf{k}$-grid, with Fermi-Dirac smearing of 10^{-5} Ry for fractional occupations. The calculation was considered to be converged only if the total energy difference between consecutive iterations within the self-consistent field cycle was smaller than the threshold value of 10^{-9} Ry.

The supercells were initially preoptimized with VASP[8] in the absence of chalcogen vacancies. For the exchange-correlation functional a local density approximation (LDA) was employed, with a basis set energy cut-off of 600 eV. The self-consistent charge density for the geometry relaxations was converged in a $6 \times 6 \times 1$ $\mathbf{k}$-grid as well. The supercells were subsequently relaxed, fixing only the position of the supercell lattice vectors and optimizing the position of the TMDC atoms within the supercell after the chalcogen atom was removed. The position of the atoms was relaxed until all components of the forces were smaller than a threshold value of 10^{-3} Ry/a_0. This second optimization was done with Quantum Espresso, using PBE and the van-der Waals corrected functional `vdw-df-09`[9–11] to properly account for changes in the interlayer separation.

GW. Using the DFT wavefunctions and energies as a starting point, we computed the corrected quasi-particle energy spectrum by performing a one-shot non-self-consistent GW calculation (G_0W_0). Our GW calculations were performed with the package BerkeleyGW, including spin-orbit interaction[12–15]. The dielectric function was obtained with the generalized plasmon-pole model of Hybertsen-Louie[16]. We employed a cut-off of 5 Ry in the dielectric screening and a total of 2499 states for the summation over the occupied and unoccupied states. We used a non-uniform neck subsampling scheme to sample the Brillouin zone close to $|\mathbf{q}| \to 0$ and speed up the convergence with respect to the $\mathbf{k}$-grid sampling[17]. Within this scheme, we considered a $6 \times 6 \times 1$ uniform $\mathbf{q}$-grid and 10 additional $\mathbf{q}$-points around $\mathbf{q} = \mathbf{0}$. A truncated Coulomb interaction was considered in the perpendicular direction to the heterostructure to prevent spurious interactions between the periodic replicas in this direction[18]. This set of parameters yields converged quasiparticle gaps within 100 meV.

BSE. To study the excitonic features, we solved the Bethe-Salpether equation (BSE)[13,19]

$$(E_{c\mathbf{k}} - E_{v\mathbf{k}})A^S_{vc\mathbf{k}} + \sum_{v'c'\mathbf{k}'} K^{\text{eh}}_{vc\mathbf{k};v'c'\mathbf{k}'} A^S_{v'c'\mathbf{k}'} = \Omega_S A^S_{vc\mathbf{k}}, \tag{S1}$$

where $E_{c\mathbf{k}}$ (resp. $E_{v\mathbf{k}}$) are the quasiparticle energies of the conduction (resp. valence) bands, $K^{\text{eh}}_{vc\mathbf{k};v'c'\mathbf{k}'} = \langle vc\mathbf{k}|\hat{K}^{\text{eh}}|v'c'\mathbf{k}'\rangle$ are the matrix elements of the electron-hole interaction kernel, defined from the addition of an attractive screened direct and a repulsive bare exchange Coulomb contributions, Ω_S is the exciton energy and $A^S_{vc\mathbf{k}}$ is the amplitude of the exciton state $|\Psi^S\rangle$. This equation sets an eigenvalue problem, $\hat{H}^{\text{BSE}}|\Psi^S\rangle = \Omega_S|\Psi^S\rangle$, where the matrix elements of the BSE Hamiltonian in the electron-hole basis are given by

$$H^{\text{BSE}}_{cv\mathbf{k};c'v'\mathbf{k}'} = (E_{c\mathbf{k}} - E_{v\mathbf{k}})\delta_{c,c'}\delta_{v,v'}\delta_{\mathbf{k},\mathbf{k}'} + K^{\text{eh}}_{vc\mathbf{k};v'c'\mathbf{k}'}. \tag{S2}$$

This representation of the BSE assumes that the (real-space) direct exciton wavefunction is described as the coherent superposition of electrons and holes at each $\mathbf{k}$-point,

$$\langle \mathbf{r}_e, \mathbf{r}_h|\Psi^S\rangle = \sum_{vc\mathbf{k}} A^S_{vc\mathbf{k}} \psi^*_{v\mathbf{k}}(\mathbf{r}_h)\psi_{c\mathbf{k}}(\mathbf{r}_e), \tag{S3}$$

with $\psi_{c\mathbf{k}}(\mathbf{r}_e)$ being the spinor wavefunction describing the electron at position $\mathbf{r}_e$ with conduction band quantum number c and crystal momentum $\mathbf{k}$ (correspondingly, $\psi_{v\mathbf{k}}(\mathbf{r}_h)$ is the spinor wavefunction describing a hole at position $\mathbf{r}_h$ and characterized by the valence band quantum number v and same crystal momentum $\mathbf{k}$).

Eq. (S1) was solved using the BerkeleyGW package[12–14]. The matrix elements were computed on a Monkhorst-Pack $9\times9\times1$ $\mathbf{k}$-grid and the result interpolated to a uniform $27\times27\times1$ $\mathbf{k}$-grid that we employ in any subsequent analysis and calculation. We employed the Tamm-Dancoff approximation and evaluated the Coulomb interaction kernel for all possible transitions between pairs of bands $(n,m) \to (n',m')$, with an energy cut-off of 5 Ry for the dielectric matrix within the electron-hole kernel matrix elements. We considered for the main paper a total of 28 bands (14 valence and 14 conduction bands) in the absorption calculations, which include both the defect bands as well as all the relevant low energy pristine valence and conduction bands of the heterobilayer. These parameters converged the calculated excitonic spectra within 100 meV for the defect-dominated subgap features and

below 10 meV for the most prominent absorption resonances in the region where intralayer non-defect TMDC excitons become more relevant. In the absorption calculation, we also avoid the heavy calculation of the $\mathbf{q}$-shifted wavefunctions on the interpolation grid by evaluation of the matrix elements of the velocity operator, $\hat{\mathbf{v}}$, instead of the momentum, $\mathbf{p} = \mathbf{i}\nabla$. This involves neglecting terms in the sums proportional to $|\langle 0|[\hat{V}_{\mathrm{ps}}, \hat{\mathbf{r}}]|S\rangle|^2$, where $\hat{V}_{\mathrm{ps}}$ is the non-local part of the pseudopotential[12,13]. Including the non-local terms has been shown to not qualitatively change the shape of water X-ray absorption spectrum[20].

Projected density of states. We compute the layer contribution of each band to a given exciton state, first by obtaining the $\mathbf{k}$-projected density of states (DoS) of each layer, $l = \{\mathrm{WS}_2, \mathrm{Gr}\}$ from the $\mathbf{k}$-resolved projected DoS

$$g^l_{n\mathbf{k}}(E) = \sum_{\{i_A, A\} \in l} |\langle \phi^A_{i_A} | \psi_{n\mathbf{k}} \rangle|^2 \delta(E - E_{n\mathbf{k}}), \tag{S4}$$

where $|\psi_{n\mathbf{k}}\rangle$ and $E_{n\mathbf{k}}$ are the Kohn-Sham states and energies and the sum runs over atoms A and orbitals i_A of the corresponding layer l. We further normalize this quantity for each layer as $\tilde{g}(E) = g(E)/\max[g(E)]$ so that

$$\tilde{g}_{n\mathbf{k}}(E) = \tilde{g}^{\mathrm{WS}_2}_{n\mathbf{k}}(E) - \tilde{g}^{\mathrm{Gr}}_{n\mathbf{k}}(E), \tag{S5}$$

is defined in the range $[-1, 1]$. This way, $\tilde{g}_{n\mathbf{k}}(E) = -1$ corresponds exclusively to graphene contribution and $\tilde{g}_{n\mathbf{k}}(E) = 1$ exclusively to TMDC contribution.

Heterostructure decomposition. We employ Eq. (S5) to display the color of the band contributions to the exciton decomposition as well as the absorbance decomposition into intralayer and interlayer parts in the main paper. In particular, for Fig. 2 and Fig. **S5**, the contributions to a given conduction band are obtained as $\sum_{vc\mathbf{k}} \tilde{g}_{v\mathbf{k}} |A^S_{vc\mathbf{k}}|^2$ while the contributions to a given valence band result from $\sum_{vc\mathbf{k}} \tilde{g}_{c\mathbf{k}} |A^S_{vc\mathbf{k}}|^2$. The $\mathbf{k}$-resolved decomposition in Fig. 3(b) of the main paper (see also Figs. S9, S12, S13) are obtained using the same expressions but without the summation over the crystal momentum.

Absorbance. From the absorption, we compute the associated absorbance using[21]

$$A(\omega) = \frac{\omega L_z}{c}\epsilon_2(\omega), \tag{S6}$$

where ω is the photon frequency, c the speed of light, L_z the distance between the heterostructure and its periodic replicas and $\epsilon_2(\omega)$ the imaginary part of the dielectric function.

Exciton binding energy. The excitonic binding energies are calculated from the difference between the expectation values of the diagonal and the full BSE Hamiltonian,

$$\begin{aligned} E^S_{\text{bind}} &= \langle\Psi^S|\hat{H}^{\text{BSE}} - \hat{K}^{\text{eh}}|\Psi^S\rangle - \langle\Psi^S|\hat{H}^{\text{BSE}}|\Psi^S\rangle, \\ &= \sum_{vc\mathbf{k}} |A^S_{vc\mathbf{k}}|^2(E_{c\mathbf{k}} - E_{v\mathbf{k}}) - \Omega_S, \end{aligned} \tag{S7}$$

where $\hat{H}^{\text{BSE}}$ is the BSE Hamiltonian and $\hat{K}^{\text{eh}}$ the electron-hole interaction kernel; $E_{\alpha\mathbf{k}}$, the quasi-particle bands; and Ω_S, the exciton energy.

Intrinsic decay rate. To compute the intrinsic decay rate of the zero-momentum excitons we follow Refs. [22–24]

$$\gamma_S = \frac{4\pi e^2}{m^2 c}\frac{\mu_S}{A_c\Omega_S}, \tag{S8}$$

where m is the electron mass, μ_S is the BSE oscillator strength of the exciton with energy Ω_S and A_c the area of the supercell.

Pristine heterostructure. For the absorption calculation of the pristine WS_2–Gr heterostructure, we considered the initial preoptimized geometry with a vacuum distance between the periodic replicas in the out-of plane direction of ~ 13 Å. The DFT calculations have the same basis set as well as non-empirical exchange correlation functional[6] as the defected heterobilayer. We considered the same convergence parameters for the basis cut-off, **k**-grid and total energy difference between consecutive iterations in the self-consistent field cycle as well. The GW calculation was performed with the same parameters within the BerkeleyGW package[12–14] as detailed previously for the defected heterostructure. Finally, the absorption was obtained by solving the BSE equation[13,19]. We use a coarse $9 \times 9 \times 1$ **k**-grid and interpolate to a uniform $27 \times 27 \times 1$ finer **k**-grid for meaningful comparison between

our results. The energy cut-off was 5 Ry for the dielectric matrix within the electron-hole kernel matrix elements and the total number of bands was 28 (14 valence and 14 conduction bands), which include all the relevant low energy pristine valence and conduction bands of the heterobilayer.

II. ADDITIONAL RESULTS

Geometry optimization. After relaxation in the presence of the vacancy, we find that close to the defect position, the nearest-neighbor metal-metal distance decreases to $d_{\mathrm{W-W}} = 3.02\,\text{Å}$ and $d_{\mathrm{Mo-Mo}} = 3.05\,\text{Å}$. This corresponds to a reduction of about $3-4\%$ in units of the TMDC monolayer lattice constant, $\bar{d}_{\mathrm{X-X}}$. Because the supercell volume is constant during the relaxation, the TMDC layer is strained in the vicinity of the defect where the distance to the next-nearest-neighbor metallic atoms increases up to $\simeq 3.2$ Å for the functional employed here. Similar behavior is also observed for the sulfur atoms surrounding the vacant site, which rigidly follow the motion of the metallic atoms. Geometry optimization also reduces the average interlayer distance in the heterostructure by around $3-4\%$. In particular, we find a reduction of the interlayer distance to $\bar{d}_{\mathrm{inter}} = 3.29$ Å in the case of MoS_2–Gr and $\bar{d}_{\mathrm{inter}} = 3.31$ Å for WS_2–Gr for our relaxation criteria and our employed van der Waals scheme.

DFT bandstructures. For completeness, we show the bandstructures obtained using the DFT relaxed structures in the presence of spin-orbit interaction in Fig. S2.

GW. As stated in the main text, our GW results qualitatively follow the DFT band structures discussed in Ref. [25]. We summarize here for completeness the main features. In essence, the band structures of graphene and the TMDC appear mostly superimposed, with the graphene Dirac cone centered at the $\bar{\mathrm{K}}$ (and $\bar{\mathrm{K}}$') points. The Dirac point sets the charge neutrality point within the pristine TMDC band gap. The combination of the missing chalcogen atom and the symmetry of the host lattice results in four empty in-gap and two occupied spin-orbit split bands. In addition, the reduction of the original TMDC symmetry due to graphene adsorption, and the residual defect-defect interaction that results from the lattice mismatch between layers within the supercell as well as the supercell size (*i.e.* the

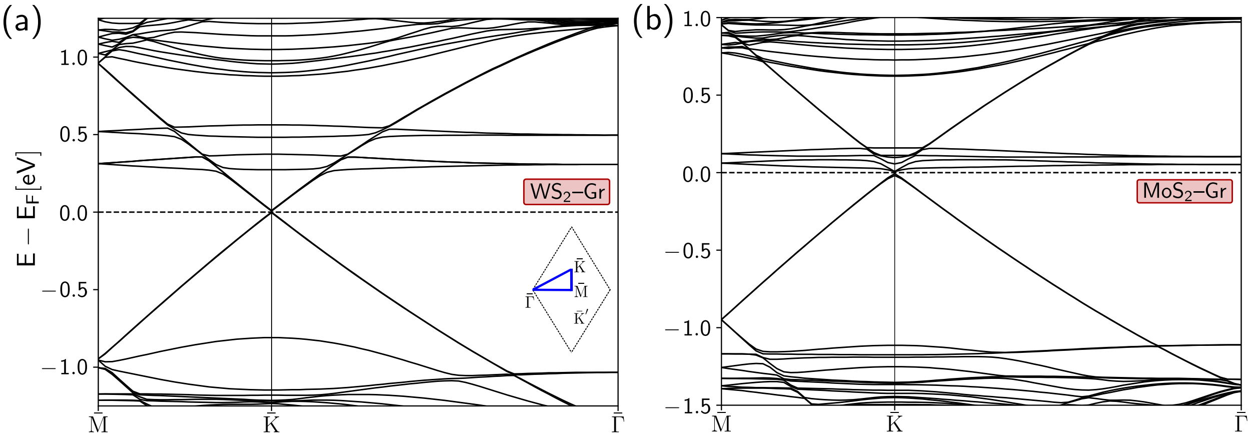

Fig. S2. Band structures of the TMDC–Gr heterostructures in the presence of spin-orbit interaction computed along the $\bar{\mathrm{M}}-\bar{\mathrm{K}}-\bar{\Gamma}$ path in the supercell (see inset) (a) WS_2–Gr heterobilayer (b) MoS_2–Gr heterobilayer.

defect density) make these bands non-degenerate and weakly dispersive. The breaking of the degeneracy is stronger in the vicinity of the $\bar{\mathrm{K}}$ and $\bar{\mathrm{K}}'$ points. Defect states also hybridize with the graphene states in certain regions of the supercell Brillouin zone. In particular, the interlayer hybridization is relatively important between the defect and the graphene bands in a ring around the $\bar{\mathrm{K}}$ and $\bar{\mathrm{K}}'$ points. This interlayer hybridization was already measured[26,27] and predicted[28] for occupied bands in MoS_2–Gr far from the charge neutrality point. Proximity induced orbital and spin-orbit effects between the TMDC and graphene also opens a gap at the Dirac cone already at the DFT level[29] ($\sim$ 1 meV). We define the Fermi energy (Dirac point) as the mid-point of this gap, $E_\mathrm{F} := E_\mathrm{D} = (E_\mathrm{val} + E_\mathrm{cond})/2$, which would correspond to the intersection point of the graphene bands if those would intersect.

Graphene adsorption and geometry relaxation within the supercell breaks the original lattice symmetry[25], lifting the degeneracy of the TMDC conduction states at the Λ and K **k**-points, both folded into the point $\bar{\mathrm{K}}$ of the supercell Brillouin zone due to lattice commensuration. For WS_2–Gr, this results in the lowest conduction band having Λ nature with K states being at higher energy already at the DFT level. The identification is performed from the pseudo-charge density, $|\Psi|^2$ at $\mathbf{k} = \bar{\mathrm{K}}$, which provides the orbital contribution, comparing to the pristine TMDC states. Screening affects more strongly the more delocalized K states (essentially due to the defect effect), shifting them higher in energy compared to the folded Λ states (by $\sim$ 0.25 eV), the latter determining thus the pristine band gap. For MoS_2–Gr, already at the DFT level the K bands are below the Λ bands by $\sim$ 0.1 eV and

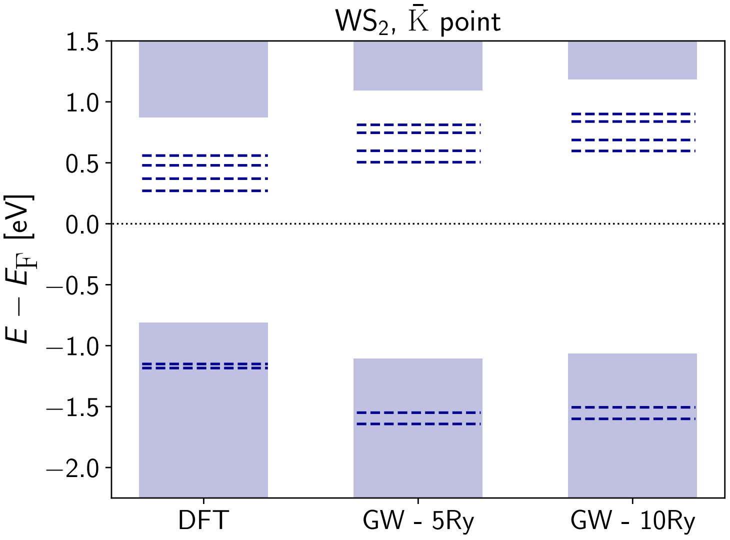

Fig. S3. DFT and GW calculated valence and conduction band energies at the $\bar{\mathrm{K}}$ point for the defected WS_2–Gr heterobilayer. We compare the DFT results with the GW calculated ones using 5 Ry and 10 Ry as dielectric cut-off.

this situation reverses at the GW level, where the K bands appear ~ 0.15 eV above. This again gives a relative shift of ~ 0.25 eV, suggesting that in both heterostructures the levels shifting results from the graphene dielectric screening.

The screening and exchange renormalize also the graphene Fermi velocity associated to the slope of the Dirac cone. In particular, we obtain an increase of the Fermi velocity of $\sim 34\%$ for WS_2–Gr and $\sim 41\%$ for MoS_2–Gr, consistent with a reported increase of the Fermi velocity of $\sim 34\%$ in Ref. [21] and slightly larger than the $\sim 18\%$ reported in Ref. [30] (both calculations for the isolated graphene monolayer). In addition, we find that for WS_2–Gr (resp. MoS_2–Gr) the Fermi energy shifts from 3.80 eV in DFT to 4.68 eV in GW with respect to the vacuum level (resp. 3.60 eV to 4.50 eV).

III. GW AND ABSORPTION CONVERGENCE CHECKS

We have checked that our GW calculations are converged with respect to the dielectric cut-off value employed in the computation of the dielectric function and the self-energy, by increasing the dielectric cut-off in the GW calculation for WS_2-Gr. Our computed GW energies are converged quantitatively up to 50 meV, see Fig. S3.

Ensuring a qualitatively **k**-grid converged absorption is crucial for understanding the

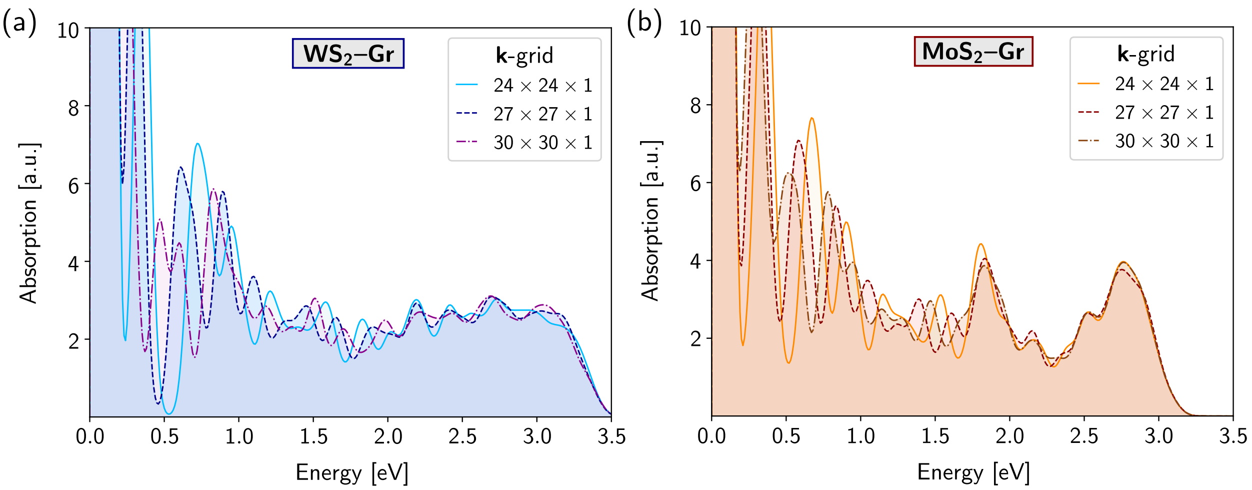

Fig. S4. Brillouin zone **k**-grid convergence of the absorption spectrum for different supercell sampling. (a) WS_2–Gr heterobilayer (b) MoS_2–Gr heterobilayer.

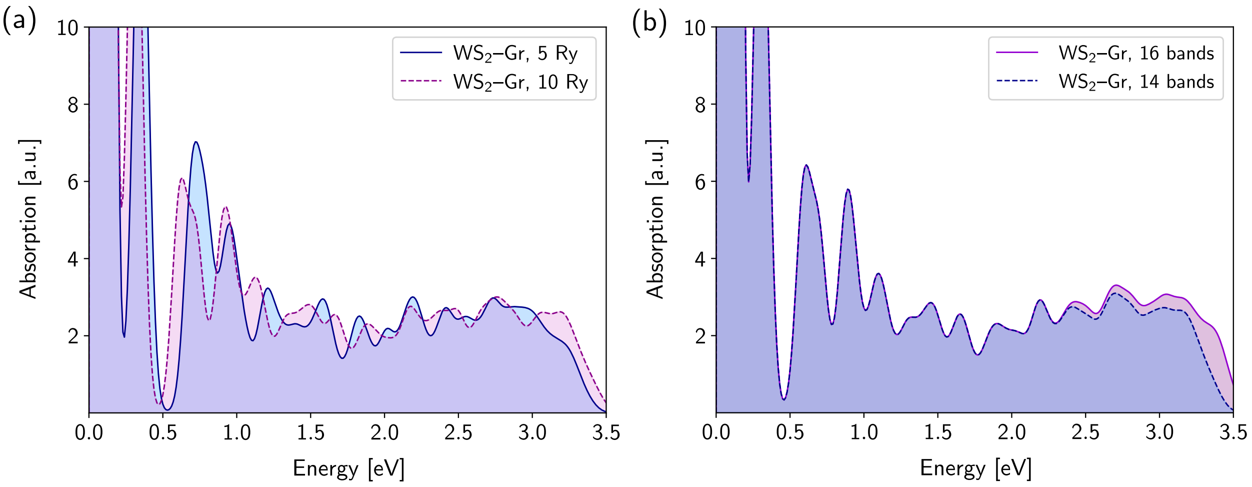

Fig. S5. Convergence of the absorption spectrum of WS_2–Gr heterobilayer with (a) the dielectric cut-off employed in the GW calculation (b) the number of bands included in the BSE calculation.

defect-induced phenomena presented in this paper. In Fig. S4, we show the absorption spectrum for different supercell Brillouin zone uniform **k**-grid samplings of the interpolated absorption grid for (a) WS_2–Gr (b) MoS_2–Gr . We find that the absorption spectra are quantitatively well converged in the visible range, especially above optical energies $\gtrsim 2$ eV for WS_2–Gr and $\gtrsim 1.75$ eV for MoS_2–Gr. In this energy range, convergence is achieved already with a $24 \times 24 \times 1$ **k**-grid up to ~ 10 meV. However, the position and height of the absorption resonances in the infrared range, especially for energies roughly below $0.3 - 0.5$ eV, are not yet fully converged. This region, which is also known to be strongly dominated by intraband graphene resonances[21], requires very dense **k**-grids for smoothness and quan-

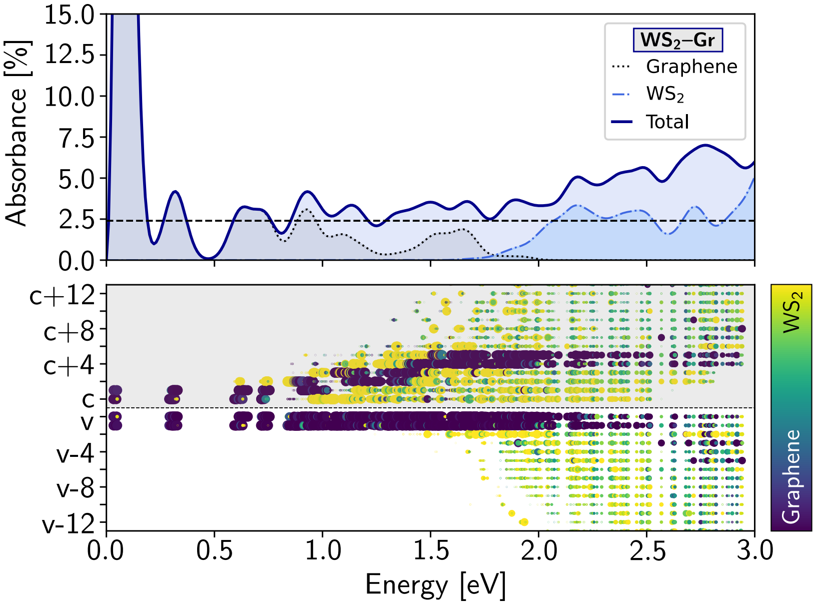

Fig. S6. Absorbance, absorbance decomposition and exciton contributions for the defected WS_2–Gr heterobilayer as described in Fig. 2 of the main text using the GW calculation with dielectric cut-off of 10 Ry.

titative convergence. In the intermediate optical range, up to $\sim 1.6 - 1.7$ eV, the results are qualitatively converged, but the main absorption features are still quantitatively dependent on the employed **k**-grid. At energies between $\sim 0.5 - 1.6$ eV, the results qualitatively agree between different **k**-grids, and, importantly, the height of the absorption resonances is stable. The importance of this observation for defect-based sub-gap features and their expected survival in the limit of dense **k**-sampling is discussed the main text. Finally, we also checked that the excitonic features discussed in the main paper are converged with respect to the dielectric cut-off employed in the GW calculation (up to 100 meV as stated above) and the number of bands included in the BSE calculation. In Fig. S5 we show in panel (a) the comparison of the absorption spectra for the defected WS_2–Gr heterobilayer with the quasi-particle energies computed with different dielectric cut-offs and in panel (b) the effect of adding four additional bands (from 28 to 32) to the BSE calculation. The absorbance and excitonic decomposition for WS_2–Gr is shown in Fig. **S6**, which corresponds to the same Fig. 2 of the main text with the larger 10 Ry dielectric cut-off for the GW energies. We find that our results are qualitatively and quantitatively converged, which strengthens our observations.

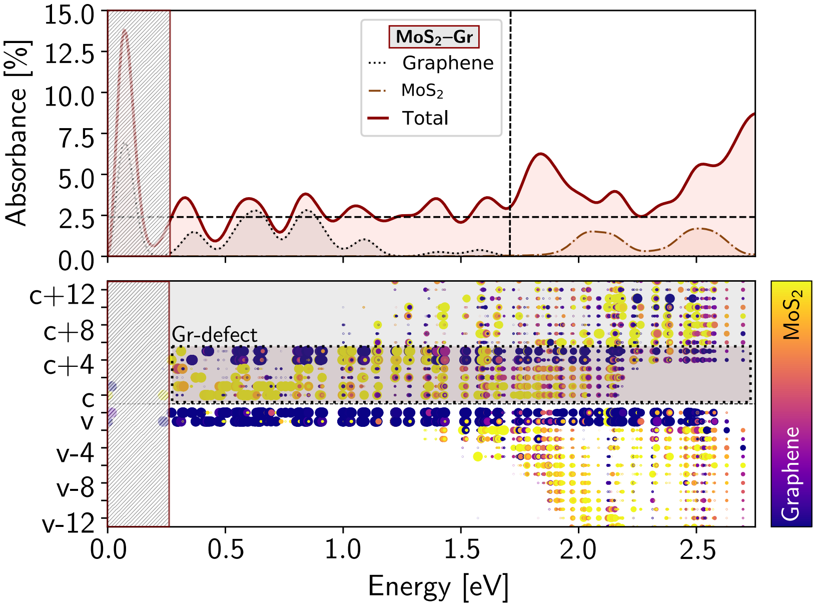

Fig. S7. Absorbance and exciton contributions for the defected MoS_2–Gr heterobilayer. (Top) Absorbance calculated along one of the main in-plane polarization directions, as well as its decomposition into graphene and MoS_2 contributions (interlayer contributions are read from the difference of the three traces). The dashed horizontal black line marks the 2.4% universal limit of graphene absorbance at infrared energies. The shaded box represents the estimated range for which we expect a smooth and monotonic spectrum dominated by graphene (instead of resonances that result from finite **k**-grid sampling). The vertical dotted line denotes the optical ranges below which excitons transition from being dominated by defect-graphene sub-gap transitions to have transitions which involve pristine TMDC bands. (Bottom) For each exciton composing the absorbance resonances, we represent the contribution of each electron and hole bands. Each dot corresponds to the band contribution to a given exciton summed over all **k** points (only bright contributions whose oscillator strength are > 5 a.u. are shown). For reasons of clarity, all dots with value $\geq 10^3$ a.u. have the same area. The color code corresponds to the layer composition of each contribution and the dotted box marks the position of the transitions towards graphene-defect empty bands.

IV. ABSORBANCE SPECTRA AND DEFECT RESONANCES IN MOS_2-GRAPHENE

As mentioned in the main text, the strong mixing that occurs in WS_2–Gr is also qualitatively similar for the case of the MoS_2–Gr interface. We show in Fig. S7, top panel, the absorbance spectrum, its decomposition into intralayer graphene, intralayer TMDC and interlayer contributions as well as the graphene universal absorbance limit at infrared energies (dashed horizontal line). Here, however, the impact of the defect bands in the absorbance is more dramatic since the defect bands are closer to the Dirac point and thus interlayer contributions affect the absorbance even at lower energies. Consequently, for MoS_2–Gr the absorbance spectrum is very different to that obtained for this TMDC with chalcogen va-

cancies in the absence of graphene layer[31,32]. In Fig. S7, bottom panel, we show the band contribution of each exciton. Note that, as compared to WS_2–Gr, here the four spin-orbit split defect bands can be identified clearly as being below the graphene Dirac cone already at optical energies ~ 0.5 eV.

V. WAVEFUNCTION DENSITIES OF RELEVANT CONDUCTION BANDS

In order to understand how the defect has such a strong impact on the absorption features, it is instructive to look at the wavefunction densities of relevant states that participate in the transitions. In Fig. S8, we show the pseudo-charge density for three sets of bands at the relevant $\bar{\mathrm{K}}$ point of the supercell Brillouin zone: (a) states with K nature, *i.e.* which have orbital contribution resembling the orbitals found in the monolayer at the K point; (b) states

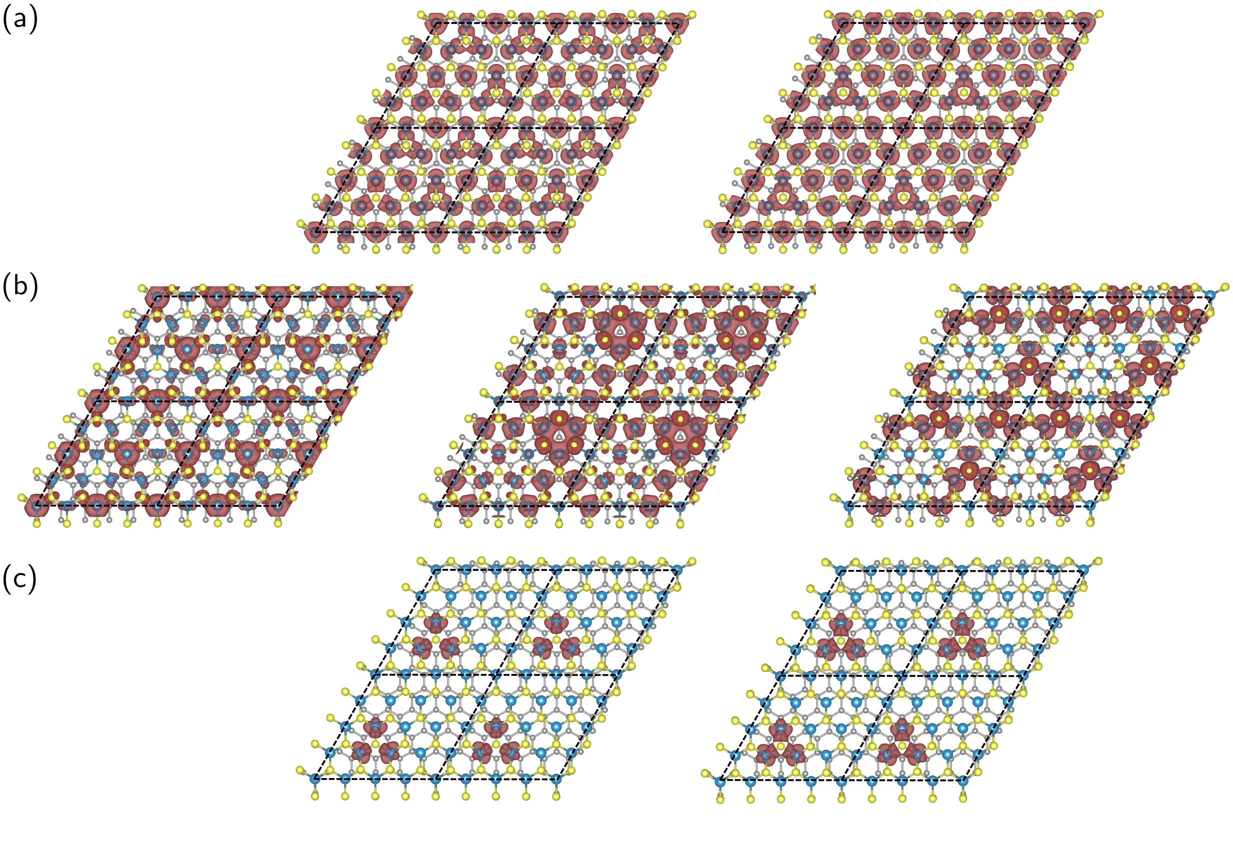

Fig. S8. (a) Top view of the Kohn-Sham pseudo-charge density, $|\Psi_{n,\mathbf{k}}(\mathbf{r})|^2$, for the conduction band states, derived from the monolayer K point. These densities are evaluated at the point $\mathbf{k} = \bar{\mathrm{K}}$ of the supercell Brillouin zone of the defected WS_2–Gr heterobilayer. (b) Same as in (a) but for the states derived from the folded Λ point, at $\mathbf{k} = \bar{\mathrm{K}}$. (c) Same as in (a) but for the two defect states with different "orbital" quantum number.[25]

originally coming from the monolayer Λ point, folded into $\bar{\mathrm{K}}$, and (c) defect states. While the states with K nature are weakly affected by the missing atom forming the vacancy, the perturbation due to the missing atom affects very strongly the Λ-like states, which have a new orbital pattern that varies at longer length scales (nanometer scale). As such, we expect these states to react differently to the dielectric screening once this is taken into account. This difference can explain therefore, as stated in the main text, why the pristine TMDC band-gap at the GW level is determined by the states shown in Fig. S8 (b) and not in (a). For comparison, we display in (c) the pseudo-densities of two defect states. These states present an orbital signature consistent with that seen in panel (b), therefore, we conclude that the behavior and shape of the Λ-derived states is exclusively determined by the vacancy.

VI. DEFECT-INDUCED EXCITON HYBRIDIZATION

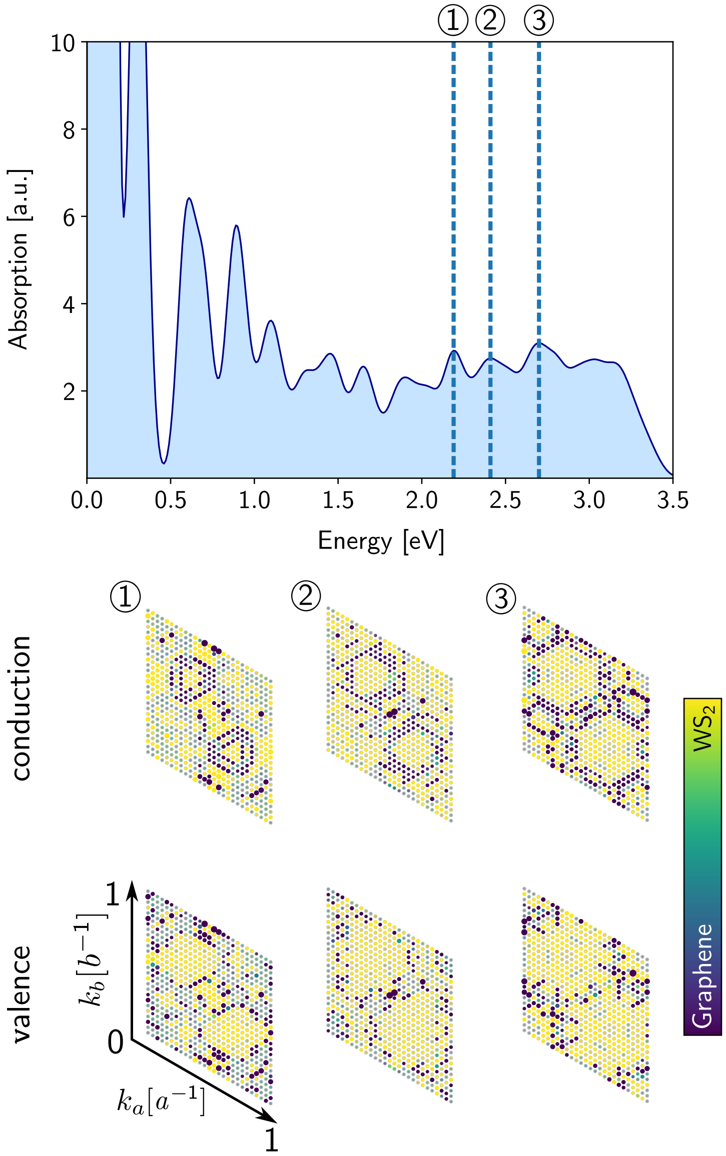

Fig. S9. Brillouin zone exciton distribution plotted for all the WS_2–Gr excitons within an energy window of ±5 meV marked in the absorption spectrum by ① (centered at 2.19 eV), ② (centered at 2.4 eV) and ③ (centered at 2.7 eV). Even a small energy window close to the center of the excitation peak shows larger hybridization between the WS_2 and the graphene layers.

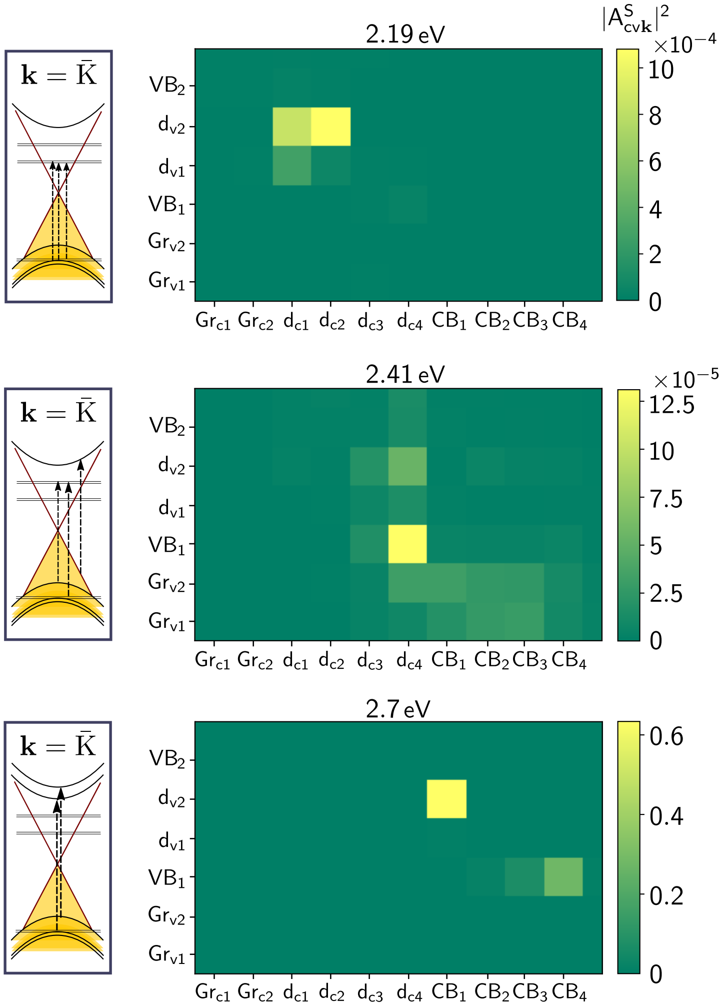

Fig. S10. Sketch of most prominent transitions and transition band diagram for the absorption peaks in Fig. S9. The excitons have all been added up within an energy window of ±5 meV.

VII. ADDITIONAL FIGURES

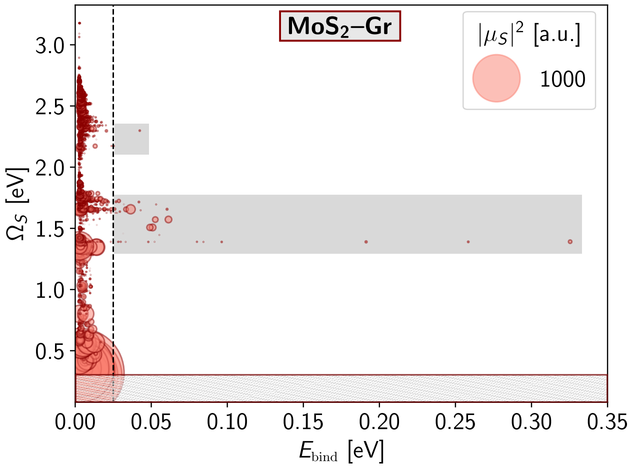

Fig. S11. Exciton energies, Ω_S, represented as a function of the binding energy, E_{bind}, for the excitons in the MoS_2–Gr heterobilayer. The binding energy is computed using Eq. (S7). We only show the excitons with $E_{\text{bind}} > 2.5$ meV (around $\sim$ 14000 out of 142884 excitons for this **k**-grid sampling and number of bands). The size of each dot is proportional to the oscillator strength (rescaled by a factor of two for visibility).

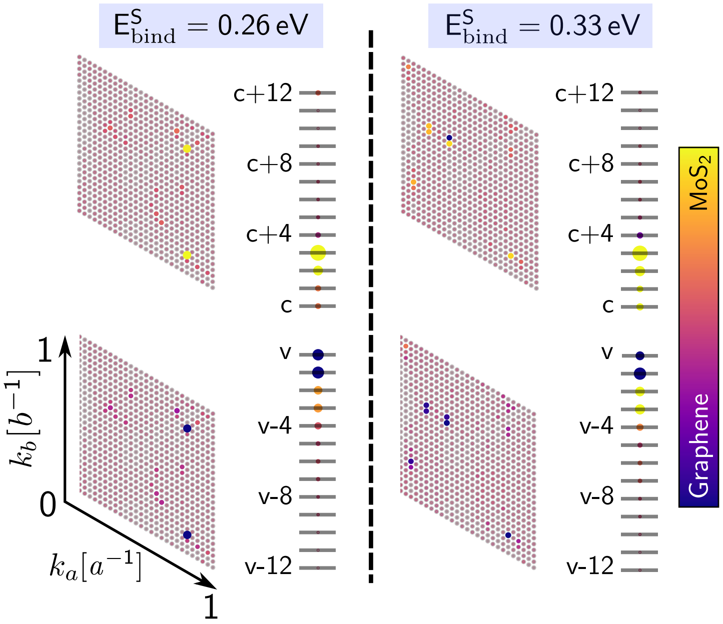

Fig. S12. Brillouin zone exciton distribution for the two most bound excitons in Fig. S11. The Brillouin zone in the top row displays $\sum_v |A^S_{cv\mathbf{k}}|^2$, while the lower row shows $\sum_c |A^S_{cv\mathbf{k}}|^2$. These two excitons present graphene-defect transitions and are largely delocalized in **k**-space.

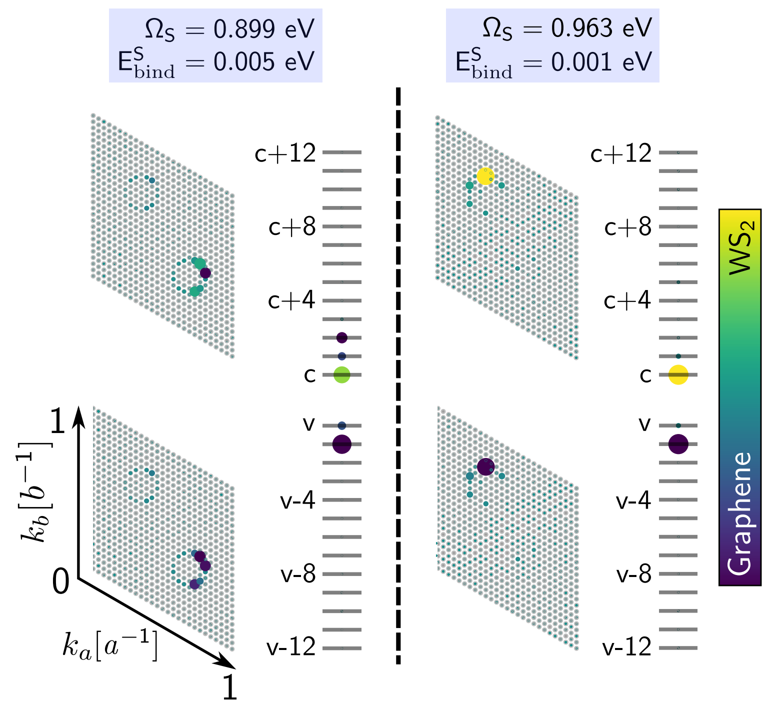

Fig. S13. Same as in Fig. S12 but for two graphene dominated excitons. Both excitons are mostly localized in the vicinity of the $\bar{\mathrm{K}}$ valleys. These excitons have a large transition dipole, with the value of μ_S being $1.5 \cdot 10^2$ a.u. and $1.2 \cdot 10^2$ a.u. respectively, but small binding energy.

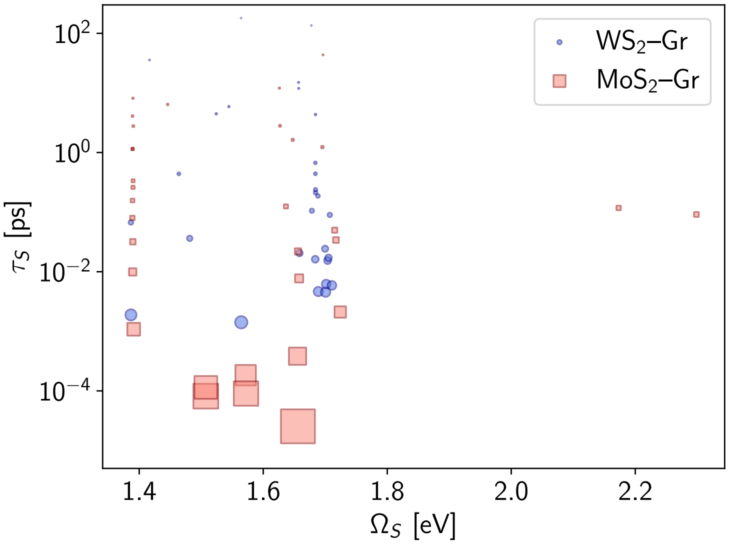

Fig. S14. Intrinsic radiative lifetimes at low temperatures for the grey excitons with binding energy larger than 25 meV, both for WS_2–Gr (blue circles) and MoS_2–Gr (red squares). The size of the points is proportional to the oscillator strength, rescaled for clarity by a factor of 20.

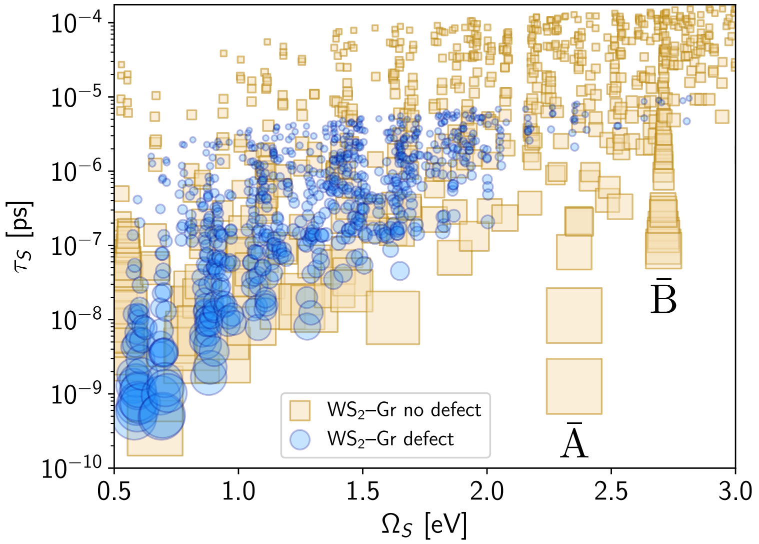

Fig. S15. Intrinsic radiative lifetimes at low temperatures for the bright excitons in the WS_2–Gr heterobilayer with and without defects. The size of the symbols is proportional to the oscillator strength μ_S, which is chosen to be larger than the threshold value of 10^1 a.u.. For clarity, we fix the value of μ_S to be 10^3 a.u if larger or equal. The grey shaded area corresponds to the region of the spectrum strongly dominated by graphene. We observe that the heterostructure without the vacancy has brighter excitons with systematically shorter lifetimes and larger oscillator strengths in the visible region, where the defected structure shows exciton quenching.

[1] N. Wakabayashi, H. G. Smith, and R. M. Nicklow, "Lattice dynamics of hexagonal MoS_2 studied by neutron scattering," Phys. Rev. B **12**, 659–663 (1975).

[2] W. J. Schutte, J. L. De Boer, and F. Jellinek, "Crystal structures of tungsten disulfide and diselenide," Journal of Solid State Chemistry **70**, 207–209 (1987).

[3] Drew Edelberg, Daniel Rhodes, Alexander Kerelsky, Bumho Kim, Jue Wang, Amirali Zangiabadi, Chanul Kim, Antony Abhinandan, Jenny Ardelean, Micheal Scully, Declan Scullion, Lior Embon, Rui Zu, Elton J. G. Santos, Luis Balicas, Chris Marianetti, Katayun Barmak, Xiaoyang Zhu, James Hone, and Abhay N. Pasupathy, "Approaching the intrinsic limit in transition metal diselenides via point defect control," Nano Letters **19**, 4371–4379 (2019).

[4] Paolo Giannozzi, Stefano Baroni, Nicola Bonini, Matteo Calandra, Roberto Car, Carlo Cavazzoni, Davide Ceresoli, Guido L. Chiarotti, Matteo Cococcioni, Ismaila Dabo, Andrea Dal Corso, Stefano de Gironcoli, Stefano Fabris, Guido Fratesi, Ralph Gebauer, Uwe Gerstmann, Christos Gougoussis, Anton Kokalj, Michele Lazzeri, Layla Martin-Samos, Nicola Marzari, Francesco Maurim, Riccardo Mazzarello, Stefano Paolini, Alfredo Pasquarello, Lorenzo Paulatto, Carlo Sbraccia, Sandro Scandolo, Gabriele Sclauzero, Ari P. Seitsonen, Alexander Smogunov, Paolo Umari, and Renata M. Wentzcovitch, "QUANTUM ESPRESSO: a modular and open-source software project for quantum simulations of materials," Journal of Physics: Condensed Matter **21**, 395502 (2009).

[5] P. Giannozzi, O. Andreussi, T. Brumme, O. Bunau, M. Buongiorno Nardelli, M. Calandra, R. Car, C. Cavazzoni, D. Ceresoli, M. Cococcioni, N. Colonna, I. Carnimeo, A. Dal Corso, S. de Gironcoli, P. Delugas, R. A. DiStasio, A. Ferretti, A. Floris, G. Fratesi, G. Fugallo, R. Gebauer, U. Gerstmann, F. Giustino, T. Gorni, J. Jia, M. Kawamura, H.-Y. Ko, A. Kokalj, E. Küçükbenli, M. Lazzeri, M. Marsili, N. Marzari, F. Mauri, N. L. Nguyen, H.-V. Nguyen, A. Otero-de-la Roza, L. Paulatto, S. Poncé, D. Rocca, R. Sabatini, B. Santra, M. Schlipf, A. P. Seitsonen, A. Smogunov, I. Timrov, T. Thonhauser, P. Umari, N. Vast, X. Wu, and S. Baroni, "Advanced capabilities for materials modelling with Quantum ESPRESSO," Journal of Physics: Condensed Matter **29**, 465901 (2017).

[6] John P. Perdew, Kieron Burke, and Matthias Ernzerhof, "Generalized gradient approximation made simple," Phys. Rev. Lett. **77**, 3865–3868 (1996).

[7] M. J van Setten, M. Giantomassi, E. Bousquet, M. J. Verstraete, D. R. Hamann, X. Gonze, and G.-M. Rignanese, "The pseudodojo: Training and grading a 85 element optimized norm-conserving pseudopotential table," Computer Physics Communications **226**, 39 – 54 (2018).

[8] G. Kresse and J. Furthmüller, "Efficient iterative schemes for ab initio total-energy calculations using a plane-wave basis set," Phys. Rev. B **54**, 11169–11186 (1996).

[9] T. Thonhauser, Valentino R. Cooper, Shen Li, Aaron Puzder, Per Hyldgaard, and David C. Langreth, "Van der Waals density functional: Self-consistent potential and the nature of the van der Waals bond," Phys. Rev. B **76**, 125112 (2007).

[10] Oleg A. Vydrov and Troy Van Voorhis, "Improving the accuracy of the nonlocal van der Waals density functional with minimal empiricism," The Journal of Chemical Physics **130**, 104105 (2009).

[11] Kristian Berland, Valentino R Cooper, Kyuho Lee, Elsebeth Schröder, T. Thonhauser, Per Hyldgaard, and Bengt I. Lundqvist, "van der Waals forces in density functional theory: a review of the vdW-DF method," Reports on Progress in Physics **78**, 066501 (2015).

[12] Jack Deslippe, Georgy Samsonidze, David A. Strubbe, Manish Jain, Marvin L. Cohen, and Steven G. Louie, "BerkeleyGW: A massively parallel computer package for the calculation of the quasiparticle and optical properties of materials and nanostructures," Computer Physics Communications **183**, 1269–1289 (2012).

[13] Michael Rohlfing and Steven G. Louie, "Electron-hole excitations and optical spectra from first principles," Phys. Rev. B **62**, 4927–4944 (2000).

[14] Meng Wu, *Spin-Orbit Coupling, Broken Time-Reversal Symmetry, and Polarizability Self-Consistency in GW and GW-BSE Theory with Applications to Two-Dimensional Materials*, Ph.D. thesis, University of California, Berkeley (2020).

[15] Bradford A. Barker, Jack Deslippe, Johannes Lischner, Manish Jain, Oleg V. Yazyev, David A. Strubbe, and Steven G. Louie, "Spinor GW/Bethe-Salpeter calculations in BerkeleyGW: Implementation, symmetries, benchmarking, and performance," Phys. Rev. B **106**, 115127 (2022).

[16] Mark S. Hybertsen and Steven G. Louie, "Electron correlation in semiconductors and insulators: Band gaps and quasiparticle energies," Phys. Rev. B **34**, 5390–5413 (1986).

[17] Felipe H. da Jornada, Diana Y. Qiu, and Steven G. Louie, "Nonuniform sampling schemes of the Brillouin zone for many-electron perturbation-theory calculations in reduced dimensionality," Phys. Rev. B **95**, 035109 (2017).

[18] Sohrab Ismail-Beigi, "Truncation of periodic image interactions for confined systems," Phys. Rev. B **73**, 233103 (2006).

[19] Michael Rohlfing and Steven G. Louie, "Electron-hole excitations in semiconductors and insulators," Phys. Rev. Lett. **81**, 2312–2315 (1998).

[20] Fujie Tang, Zhenglu Li, Chunyi Zhang, Steven G. Louie, Roberto Car, Diana Y. Qiu, and Xifan Wu, "Many-body effects in the X-ray absorption spectra of liquid water," Proceedings of the National Academy of Sciences **119**, e2201258119 (2022).

[21] Li Yang, Jack Deslippe, Cheol-Hwan Park, Marvin L. Cohen, and Steven G. Louie, "Excitonic effects on the optical response of graphene and bilayer graphene," Phys. Rev. Lett. **103**, 186802 (2009).

[22] Catalin D. Spataru, Sohrab Ismail-Beigi, Rodrigo B. Capaz, and Steven G. Louie, "Theory and ab initio calculation of radiative lifetime of excitons in semiconducting carbon nanotubes," Phys. Rev. Lett. **95**, 247402 (2005).

[23] Maurizia Palummo, Marco Bernardi, and Jeffrey C. Grossman, "Exciton radiative lifetimes in two-dimensional transition metal dichalcogenides," Nano Letters **15**, 2794–2800 (2015).

[24] Hsiao-Yi Chen, Maurizia Palummo, Davide Sangalli, and Marco Bernardi, "Theory and ab initio computation of the anisotropic light emission in monolayer transition metal dichalcogenides," Nano Letters **18**, 3839–3843 (2018).

[25] Daniel Hernangómez-Pérez, Andrea Donarini, and Sivan Refaely-Abramson, "Charge quenching at defect states in transition metal dichalcogenide–graphene van der Waals heterobilayers," Phys. Rev. B **107**, 075419 (2023).

[26] Horacio Coy Diaz, José Avila, Chaoyu Chen, Rafik Addou, Maria C. Asensio, and Matthias Batzill, "Direct observation of interlayer hybridization and Dirac relativistic carriers in Graphene/MoS_2 van der Waals heterostructures," Nano Letters **15**, 1135–1140 (2015).

[27] Debora Pierucci, Hugo Henck, Jose Avila, Adrian Balan, Carl H. Naylor, Gilles Patriarche, Yannick J. Dappe, Mathieu G. Silly, Fausto Sirotti, A. T. Charlie Johnson, Maria C. Asensio, and Abdelkarim Ouerghi, "Band alignment and minigaps in monolayer MoS_2-graphene van der Waals heterostructures," Nano Letters **16**, 4054–4061 (2016).

[28] Mit H. Naik and Manish Jain, "Substrate screening effects on the quasiparticle band gap and defect charge transition levels in MoS_2," Phys. Rev. Materials **2**, 084002 (2018).

[29] Martin Gmitra, Denis Kochan, Petra Högl, and Jaroslav Fabian, "Trivial and inverted Dirac bands and the emergence of quantum spin Hall states in graphene on transition-metal dichalcogenides," Phys. Rev. B **93**, 155104 (2016).

[30] Paolo E. Trevisanutto, Christine Giorgetti, Lucia Reining, Massimo Ladisa, and Valerio Olevano, "Ab initio *GW* many-body effects in graphene," Phys. Rev. Lett. **101**, 226405 (2008).

[31] Elmar Mitterreiter, Bruno Schuler, Ana Micevic, Daniel Hernangómez-Pérez, Katja Barthelmi, Katherine A. Cochrane, Jonas Kiemle, Florian Sigger, Julian Klein, Edward Wong, Edward S. Barnard, Kenji Watanabe, Takashi Taniguchi, Michael Lorke, Frank Jahnke, Johnathan J. Finley, Adam M. Schwartzberg, Diana Y. Qiu, Sivan Refaely-Abramson, Alexander W. Holleitner, Alexander Weber-Bargioni, and Christoph Kastl, "The role of chalcogen vacancies for atomic defect emission in MoS_2," Nature Communications **12**, 3822 (2021).

[32] Tomer Amit, Daniel Hernangómez-Pérez, Galit Cohen, Diana Y. Qiu, and Sivan Refaely-Abramson, "Tunable magneto-optical properties in MoS_2 via defect-induced exciton transitions," Phys. Rev. B **106**, L161407 (2022).
